# A Review: The Manifestations, Mechanisms, and Treatments of Musculoskeletal Pain in Patients With COVID-19

**DOI:** 10.3389/fpain.2022.826160

**Published:** 2022-03-10

**Authors:** Lijuan Wang, Na Yang, Jinfeng Yang, Shuwu Zhao, Chen Su

**Affiliations:** ^1^Department of Anesthesiology, Hunan Cancer Hospital/The Affiliated Cancer Hospital of Xiangya School of Medicine, Central South University, Changsha, China; ^2^Department of Medicine, University of South China, Hengyang, China

**Keywords:** COVID-19, musculoskeletal symptoms, myalgia, mechanisms, treatments

## Abstract

The outbreak of COVID-19 poses a serious threat to global health. Musculoskeletal (MSK) pain is the most frequent symptom in patients with COVID-19 besides fever and cough. There are limited studies addressing MSK symptoms in patients with COVID-19. This review aims to provide an overview of current studies related to MSK pain in patients with COVID-19, summarize the possible mechanisms of myalgia, and describe the current management options. In addition to acute respiratory manifestations, COVID-19 might also affect neurological systems which include skeletal manifestations and muscular injury. A possible mechanism of MSK pain and myalgia in COVID-19 may be related to the distribution of angiotensin-converting enzyme 2 (ACE-2) and the occurrence of cytokine storms. ACE-2 has been shown to be the receptor of Severe Acute Respiratory Syndrome Coronavirus 2 (SARS-COV2). Moreover, studies have shown that inflammatory cytokines could cause myalgia by inducing prostaglandin E2 (PGE2) production. In addition, it was also found that the plasma levels of IL2, IL7, IL10, IL-6, TNFα, and e lymphopenia were higher in patients with COVID-19. In general, the treatment of MSK pain in patients with COVID-19 falls into pharmacological and non-pharmacological interventions. Various treatments of each have its own merits. The role of vaccination is irreplaceable in the efforts to prevent COVID-19 and mitigates its subsequent symptoms.

## Introduction

In December 2019, COVID-19, caused by Severe Acute Respiratory Syndrome Coronavirus 2 (SARS-COV2), occurred in China and rapidly spread to every corner of the world. The virus posed a serious threat to global health and the world economy. The high infectivity of the initial strain and the emergence of new variants with higher transmissibility increased immune escape and reduced the efficacy of vaccines, thus increasing international spread (1). As of March 28, 2020, the virus has spread to over 200 countries (2). Since the outbreak, more than 224 million cases and 4.6 million deaths have been confirmed worldwide (3). Unfortunately, this number continues to rise. The pandemic has led to tremendous economic and social instability. An estimated number of $77 billion to $2.7 trillion of economic losses are occurring world wide (4). In light of this, the Chinese government has allocated about RMB 110.48 billion for epidemic prevention and control in 2020.

Previous studies have confirmed that the most common symptoms of COVID-19 are fever (98%), cough (76%), fatigue, or myalgia (44%). Less common symptoms include sputum production (28%), headache (13%), taste impairment (21%), smell impairment (19%), hemoptysis (5%), and diarrhea (3%) (5–9). The common characteristics of the virus appear to change with its frequent mutations. Real-time reverse transcriptase-polymerase chain reaction tests (RT-PCR) for the detection of the SARS-CoV-2 viral nucleic acid is the gold standard for diagnosis (10).

Historically, it has been reported that musculoskeletal (MSK) pain is the greatest contributor to the global burden of disability. It is also a major reason for people to seek healthcare (11, 12). Recently, MSK symptoms (36%) have been reported as one of the most common symptoms among patients with COVID-19 aside from fever (82%) and cough (61%) (13). In addition, MSK pain has been frequently reported by patients suffering from long-term post-COVID-19 sequelae (14, 15). Quality of life for patients suffering from post-COVID-19 syndrome and fatigue is significantly worsened by the presence of MSK pain. However, MSK symptoms in patients with COVID-19 have not attracted the same level of attention from the medical profession and scientists compared to other symptoms, such as fever, cough, anosmia, and ageusia. The intent of this review is to describe and present the current science related to MSK pain associated with COVID-19.

## Musculoskeletal Manifestations of COVID-19

COVID-19, as well as other coronaviruses, such as Severe Acute Respiratory Syndrome Coronavirus (SARS-COV) and Middle East Respiratory Syndrome Coronavirus (MERS-COV), cause respiratory symptoms ranging from mild to life-threatening (7, 16). Coronaviruses can have extensive systemic effects including attacking the neurological, urinary, cardiovascular, gastrointestinal, hematological, hematopoietic, and reproductive systems (17, 18). Neurologic manifestations of COVID-19 can be divided into 3 categories: central nervous system manifestations, peripheral nervous system manifestations, and skeletal muscular symptoms. Central nervous system manifestations include headache, dizziness, impaired consciousness, acute cerebrovascular diseases, and seizures. Peripheral nervous system manifestations include the loss of taste and smell, as well as neuralgias (8, 19). The most common MSK symptoms include fatigue, myalgias, arthralgias, and back pain (20, 21).

Xu and Huang et al. reported that myalgia or fatigue were the most common symptoms at the onset of COVID-19 and were observed in up to 30–50% of patients (7, 22, 23). Myalgias, arthralgias, and fatigue are frequently present in patients with COVID-19 and have been reported in as many as 90% of them. Interestingly, there is no reported difference in the incidence of fatigue and myalgias between mild and severe cases of COVID-19 (24). The incidence of arthralgia is inversely related to the severity of COVID-19, with 49.5% of patients with mild disease and 29.8% of patients with severe cases reporting its presence (25). In general, the presence of these symptoms increases the likelihood of a COVID-19 diagnosis (26). In a 2021 report, 25% of patients with COVID-19 presented with only MSK symptoms and fever and without respiratory symptoms (27). A recent study reported that the back was the most common region of pain for patients seen in an outpatient clinic (21). In the current pandemic, it is important that clinicians be aware of patients who could be presenting atypical or nonspecific symptoms. Awareness of these symptoms and atypical presentations could help improve the speed of an accurate initial diagnosis, reducing transmission.

Post-COVID-19 syndromes develop during or after COVID-19. These syndromes last for more than 12 weeks and are a diagnosis of exclusion (14). Post-COVID-19 syndromes are being reported more frequently and often include MSK symptoms. Particularly, joint and muscle pain are typically reported by those having post-COVID-19 syndromes (14). A meta-analysis published by Fernández-de-Las-Peñas et al. noted that the incidence of musculoskeletal pain in patients with post-COVID-19 syndrome in their first year after infection approached 10% (28). The prevalence of post-COVID-19 MSK pain has been reported to reach 38% at 7 months after hospitalization (15).

When patients developed symptoms of focal muscle pain and fatigue, clinicians should consider the diagnosis of rhabdomyolysis.

Rhabdomyolysis, is a well-known life-threatening disorder that has been reported to be a late complication of COVID-19 (29). Patients developing significant focal muscle pain, especially in the presence of myoglobinuria should be considered for a diagnosis of rhabdomyolysis. Rhabdomyolysis is conclusively diagnosed by serologic testing, namely, by determining the level of serum creatine phosphokinase (CK) (30). Rapid clinical recognition and treatment of rhabdomyolysis associated with COVID-19 can reduce the risk for serious complications and death.

## The Possible Mechanisms of Myalgia in COVID-19

The angiotensin-converting enzyme 2 (ACE-2) has been shown to be the functional receptor of the SARS-COV (31). SARS-CoV-2 (COVID-19) was named for its similarity to the SARS virus. Genomic analysis revealed that the novel SARS-COV-2 and SARS were highly homologous, sharing 79.5% of the same sequences (32, 33). Zhou et al. reported that ACE-2 was also the main receptor of COVID-19 (31, 33). The spike protein on COVID-19 uses the ACE-2 receptor to enter cells (34). The ACE-2 receptor is widely distributed in the body, especially in the lungs and epithelial cells of the small intestine (6, 35). The expression of ACE-2 in lungs and small intestine may help explain the routes of infection *via* the respiratory tract and fecal-oral route. ACE-2 receptors are also found in nerve tissue and the endothelial cells of many other organs. The wide distribution of ACE-2 may account for the multisystem involvement of COVID-19. Related to myalgias, ACE-2 is found in skeletal muscle as well as the central nervous system. Yamagata et al. found that the angiotensin-converting enzyme (ACE)/angiotensin (Ang) II/Ang II type 1 (AT1) receptor pathway promotes spinal pain transmission, but the ACE2/Ang (1-7) receptor pathway could alleviate hyperalgesia by inhibiting phosphorylation of p38 MAPK (36, 37). Therefore, it is possible that COVID-19 affects the ACE2-positive cells in the human spinal cord, causing the reduction of ACE-2 and Ang (1–7). The reduction of Ang (1–7) caused an increase in p38 MAPK phosphorylation, which results in hyperalgesia (34).

Another mechanism of MSK pain may be related to a cytokine storm. Among cytokine storms, interleukin-6 (IL-6) is a pivotal element of cytokine storm (38). The admission level of IL-6 is considered as a predictor to determine whether patients with COVID-19 need mechanical ventilation (39). In addition, what makes the preceding statement true is that IL-6 receptor blockade-tocilizumab is proven to be effective in patients with COVID-19 pneumonia and elevated IL-6 (40, 41). IL-6 acts on musculoskeletal tissue and can cause myalgias by inducing prostaglandin E2 production (42, 43). Besides IL-6, patients with severe cases of COVID-19 have higher plasma levels of other elements of cytokine storms, including IL-2, IL-7, IL-8, IL-10, and tumor necrosis factor-α (TNF-α) (7). These findings are supported by additional studies which also indicate that those cytokine storms are related to disease severity and mortality in patients with COVID-19 (44). Rapid replication of SARS-CoV-2 is responsible for the production of mass proinflammatory cytokines (45). Therefore, there is the strong possibility that COVID-19 myalgia pathogenesis refers to the cytokine storm.

Further studies have suggested that direct injury from the virus might be another mechanism for myalgias in patients with COVID-19 (46). However, Sansin Tuzun et al. found that CK, an indicator of muscle damage, was not associated with the development of myalgia and proposed that myalgia in patients with COVID-19 may mainly be related to functional impairment as opposed to true tissue damage (25). Immune dysfunction and hypoxic injury have been proposed as mechanisms that account for myalgias in viral infections (47–51). Whether these pathogenesis account for the manifestations of COVID-19 needs to be further investigated. Pain pathogenesis is multifactorial. The mechanisms of MSK symptoms in COVID-19 are still under investigation.

## Treatment

Musculoskeletal (MSK) pain, as a symptom of COVID-19, causes significant suffering and reduces the quality of life of many patients. Treatment of pain in patients with COVID-19 should not be neglected. Conventional treatments for myalgia are varied, including pharmacological agents, non-pharmacological interventions, including psychosocial interventions and exercise therapy, and complementary therapies, such as acupuncture and Shiatsu (52). These three therapeutic approaches have varying levels of efficacy. The most commonly used drugs for treating MSK pain are opioids, non-steroidal anti-inflammatory drugs (NSAIDs), and corticosteroids. Due to pathological changes and clinical manifestations of COVID-19, the application of these drugs for the treatment of myalgia in patients with COVID-19 might subtly produce different results.

Currently, there are no specific drugs for the treatment of MSK pain in patients with COVID-19.

## Pharmacological Treatments

Opioids are one of the most potent and oldest classes of drugs for the treatment of acute and chronic pain. However, opioids, especially morphine and fentanyl, have been demonstrated to induce immune suppression (53, 54). The immune system of patients infected with COVID-19 can be significantly abnormal (55, 56). Aside from comorbidities, such as age, diabetes, and HTN, chronic opioid use may augment the risk of immunosuppression and worsen a COVID-19 infection (57). Clinicians should consider the possibility of increased susceptibility to COVID-19 infections and other secondary infections caused by immunosuppression associated with opioids. Furthermore, opioid addiction and misuse have caused a public health crisis, resulting in death and major social and economic consequences separate from the pandemic (58). Indirectly, the COVID-19 pandemic has already worsened the catastrophic opioid crisis (59). In comparison, buprenorphine seems to be safer to use in immunocompromised patients who are susceptible to infection (60). William et al. noted that buprenorphine is safe and supported by new dose titration protocols, reducing concerns about opioid withdrawal (59, 61). Fever in patients with COVID-19 can cause flushing and vasodilation, possibly increasing the absorption of opioids if transdermal patches are used to treat pain (46). Caution should generally be used when applying transdermal opioid patches in febrile patients. Opioids should not be the first choice of treatment for any patient with MSK pain, including those with COVID-19. If an opioid is considered appropriate for the treatment of moderate-to-severe MSK pain, buprenorphine may be a preferred option for several reasons, including its reduced level of immunosuppression.

Non-steroidal anti-inflammatory drugs (NSAIDs) are commonly used for controlling fever and MSK pain. NSAIDs exert their analgesic effect through the peripheral inhibition of the synthesis of peripheral prostaglandins by the enzyme cyclo-oxygenase (COX) (62). In a clinical study, Covi et al. found that prostaglandins stimulated the antihypertensive effects of ACE inhibitors. In contrast, anti-inflammatory drugs decreased this effect by inhibiting prostaglandin synthetase (63). It has been speculated that NSAIDs could increase ACE tissue levels and indirectly worsen the severity of COVID-19. Therefore, it should be avoided. Moreover, a multi-source analysis from the Regional Pharmacovigilance Centers (CRPV) suggests that NSAIDs, such as ibuprofen and ketoprofen, would aggravate bacterial infections, especially pulmonary infections (64). However, The European Medicine Agency announced that NSAID use was unrelated to worsening of COVID-19 infections. There is currently no clear contraindication for using NSAIDs in patients with COVID-19 (65). According to European Union's (EU) National Treatment Guidelines, patients with COVID-19 can continue to take NSAIDs (like ibuprofen) in the lowest effective dose for the shortest required time (66). There is no clear evidence demonstrating a relationship between NSAIDs and disease prognosis in patients with COVID-19. Besides NSAID, paracetamol may be a good option as it has both antipyretic and analgesic effects. The EU National Treatment Guidelines recommend paracetamol as the first option for symptomatic treatment of mild symptoms of COVID-19, especially for fever and myalgias (65).

Corticosteroids have been used to treat painful MSK disorders, such as osteoarthritis, partially due to their anti-inflammatory action and rapid onset (67). However, higher doses and long-term use may lead to significant side-effects which include osteoporosis, osteonecrosis, myopathy, immunosuppression, and myalgias. These adverse effects would clearly negatively impact the well-being of patients (68). Corticosteroids, in a dose-dependent manner, were implicated as a cause of osteonecrosis in patients with SARS (69). However, a study including seven international clinical trials demonstrated that corticosteroids produced a 20% relative reduction in the risk of death in critically ill patients with COVID-19 (70). Hence, in September 2020, the WHO updated its guidance that recommends the use of systemic corticosteroids to treat patients with severe COVID-19 or in the state of refractory shock (46, 71). For patients with mild COVID-19, the WHO discourages the use corticosteroids as a first-line treatment. Corticosteroids do not appear to provide many benefits and may even be harmful (71). With these considerations in mind, it is recommended to avoid the use of corticosteroids alone in treating COVID-19 MSK pain.

## Non-Pharmacological Interventions

During a pandemic, lockdowns and quarantining help reduce the spread of disease by restricting mass movements (72). Protective measures may also cause a number of harmful effects, including psychological stress and a worsening of musculoskeletal symptoms that is especially notable in women (73). Psychological stress can originate from long periods of isolation, fear of infection, lack of daily supplies, and financial losses (74). An online survey from China indicated that 16.5 to 28.8% of the respondents living through the pandemic reported moderate-to-severe depression and anxiety, the presence of which was significantly associated with physical symptoms, such as myalgias (75). Hence, the establishment of a social-psychological-biomedical treatment model and the adoption of a combination treatment of psychological and drug therapy may be ideal.

Physical inactivity caused by the social restrictions in place may worsen MSK pain (72). Regular physical exercise plays a significant role in modulating the immune system. Exercise stimulates the release of pro- and anti-inflammatory cytokines and increase of lymphocyte circulation, as well as cell recruitment. Regular physical activity is also associated with a lower incidence of mortality in patients with viral infections (76). Other studies have shown that regular and moderate exercise help to reduce the risk for upper respiratory tract infections in general (77). Another benefit of physical exercise is the overall reduction of communicable diseases, including viral pathologies (78). Exercise therapy has also been proven to be beneficial for pain and quality of life in patients with myalgia (52). An emphasis on maintaining exercise during the COVID pandemic will help improve overall health and potentially reduce other complications.

## Telemedicine

Telemedicine is emerging as a valuable technology to both patients and health care providers. It can be used to reduce the risk of the spread of COVID-19 while providing ongoing care to patients with chronic pain and/or COVID-19. Telemedicine removes the need for patients to travel to healthcare centers and eliminates physical contact between patients and medical workers, thereby reducing the risk of COVID-19 transmission. Telemedicine can facilitate consultation, diagnosis, monitoring, and mentoring through virtual encounters and e-visits (79, 80).

Commonly used instruments for pain-intensity assessment are the Visual Analog Scale (VAS), Graphic Rating Scale (GRS), and Verbal Rating Scale (VRS). Other instruments to measure pain include Pain-O-Meter and McGill Pain Questionnaire (MPQ) (81). All of these tests can be conducted *via* telemedicine/eHealth interviews (82). Patients with mild-to-moderate post-COVID-19 pain, who have returned home and are continuing pain treatment may also benefit from telemedicine support (82). Telemedicine can also help facilitate follow-up visits of patients after discharge and allows patients to maintain the continuity of care while remaining at home (82, 83). Further, during the COVID-19 epidemic, patients who are prescribed opioids can be closely monitored without the need for frequent office visits. It is hoped that this kind of intense monitoring will help reduce opioid abuse (84).

Telemedicine has some obvious shortcomings. The use of audio and video cannot replace physical examination which plays a significant role in clinical evaluation. Depending on their social situation, telemedicine could reduce privacy for some patients (85). There are also a series of other medico-legal issues that can arise with its use, such as practicing across territorial lines (85, 86). In general, telemedicine is a good choice for pain patients and clinical workers during the COVID-19 pandemic or in the presence of an infectious disease.

## Vaccine

Prevention is at the heart of public health. Safe and effective vaccines are critical to control the occurrence and prevalence of COVID-19. It is hoped that through vaccination and natural immunity, the pandemic will eventually end. Currently, there are 8 vaccines that have been shown to be safe and efficacious and are approved for use by the WHO (87). In clinical trials, some vaccines have been reported to have efficacy exceeding 90% against COVID-19 (88). Fundamentally, interrupting the transmission of COVID-19 is the best method of reducing the incidence of COVID-19-induced MSK pain. COVID-19 vaccine breakthrough infections, defined as an infection occurring in fully vaccinated people, appear to be associated with less severe MSK pain compared to patients with MSK from COVID-19 who are unvaccinated (89–91). However, literatures about clinical features of breakthrough cases are limited, thereby emphasizing the need for such research. Recently, Fikadu G et al. reported that levels of antibody and variant cross-neutralization were increased after COVID-19 vaccine breakthrough infection (91). Patients with breakthrough infections might develop stronger immunity, especially when it occurs in the presence of prior vaccination. In addition, the possibility of variant infections after vaccine breakthrough infection might further decline. Hence, it is still worthwhile to promote vaccination.

## Conclusion

COVID-19 poses a great threat to global, economic, and social health. MSK pain is the most frequent symptom reported by COVID-19 patients and is one of the greatest contributors to the global burden of disability which affects daily life. At the onset of a COVID-19 infection, myalgia or fatigue may have a high degree of specificity for the disease and is one of the most common reported symptoms. The presence of these symptoms can help clinicians in making an accurate initial diagnosis. Patients who have recovered from COVID-19 often experience MSK symptoms, with fatigue, myalgia, arthralgia, and back pain being the most reported. The ACE2/Ang (1–7) receptor pathway and cytokine storms may play a role in the possible mechanism of myalgia seen in COVID-19 disease. Treatments for myalgias may include pharmacological treatments and non-pharmacological interventions. Paracetamol is recommended as the first option for the symptomatic treatment of COVID-19, especially for patients with fever and myalgia. For other pharmacological management, opioids could be considered, taking into consideration appropriate selection, dosage, duration, and tracking, along with appropriate discontinuation and evaluation of risks. Opioids should not be considered as first-line treatment for COVID-19-induced MSK pain. If opioids are considered for moderate-to-severe MSK pain, due to their limited effects on immunosuppression, buprenorphine is highly suggested to be the preferred agent. NASIDS, in general, are the most common choice for the treatment of MSK pain and are proven to be useful in patients with COVID-19. Systemic corticosteroids should only be used in patients with severe COVID-19. Non-pharmacological interventions can play a significant role in the treatment of myalgia in patients with COVID-19. Telemedicine, which is conducive to reducing the spread of COVID-19 while guaranteeing timely and effective treatment for the pain patients, may prove to be very useful. Finally, prevention of the virus and infection reduction is probably the most important method to reduce MSK-related symptoms.

Further research is required to fully understand the mechanisms causing myalgia and MSK pain from COVID-19. Moreover, additional studies are needed to reveal the optimal treatment regimens for patients suffering from COVID-19-related symptoms.

## Author Contributions

LW and NY conceived and wrote the article. JY, SZ, and CS revised and reviewed the article. All authors contributed to the article and approved the submitted version.

## Funding

This work was supported by grants from the National Natural Science Foundation of China (No. 81901141) and the Scientific Research Project of Hunan Provincial Health Commission (202204114480).

## Conflict of Interest

The authors declare that the research was conducted in the absence of any commercial or financial relationships that could be construed as a potential conflict of interest.

## Publisher's Note

All claims expressed in this article are solely those of the authors and do not necessarily represent those of their affiliated organizations, or those of the publisher, the editors and the reviewers. Any product that may be evaluated in this article, or claim that may be made by its manufacturer, is not guaranteed or endorsed by the publisher.
